# Supplementary material for: Evidence supported by Mendelian randomization: impact on inflammatory factors in knee osteoarthritis
Source: Front Med (Lausanne). 2024 May 28;11:1382836. doi: 10.3389/fmed.2024.1382836 (PMC11165061; doi:10.3389/fmed.2024.1382836)
Supplement: Supplementary file 1 [file Data_Sheet_1.ZIP › data/GCST90274848_5e6/848.forest.pdf]

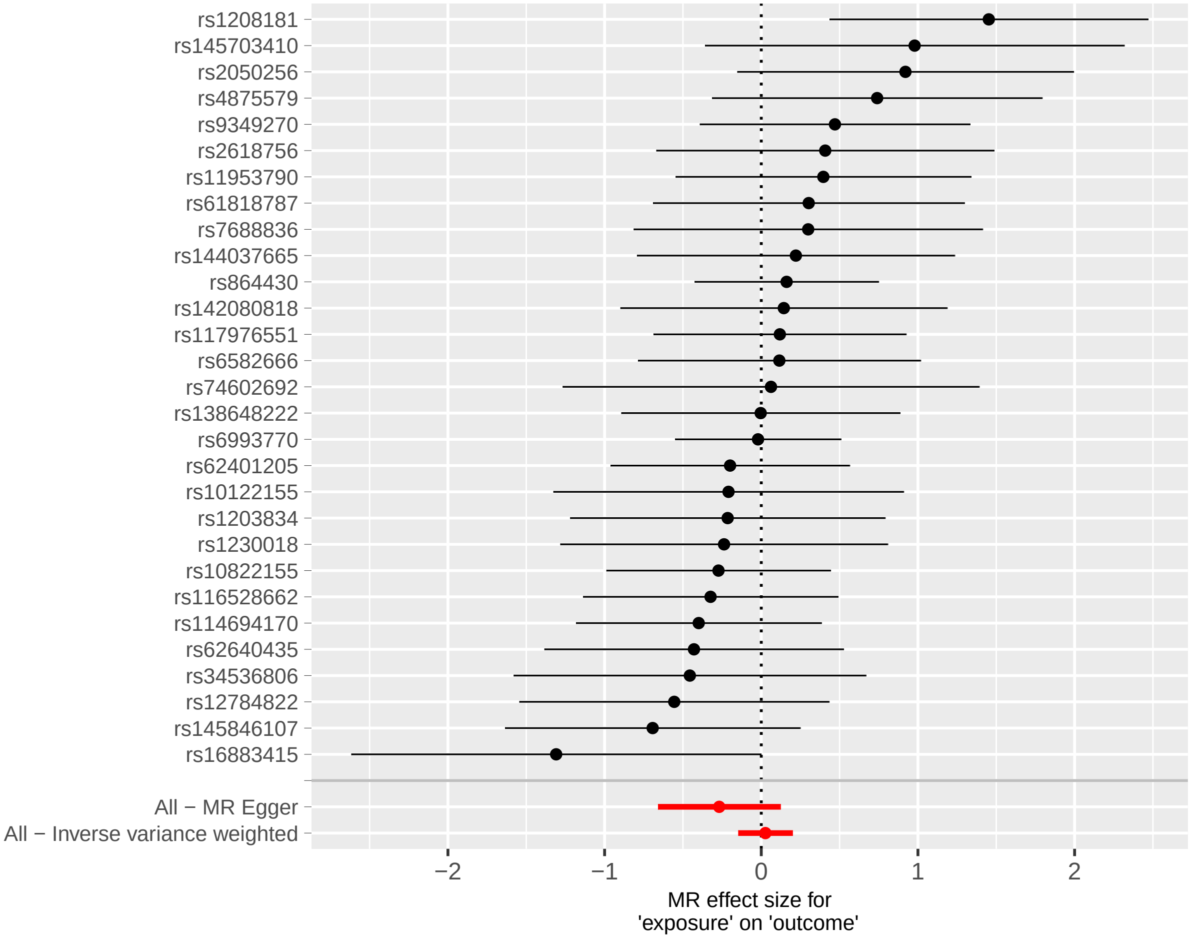
rs1208181
rs145703410
rs2050256
rs4875579
rs9349270
rs2618756
rs11953790
rs61818787
rs7688836
rs144037665
rs864430
rs142080818
rs117976551
rs6582666
rs74602692
rs138648222
rs6993770
rs62401205
rs10122155
rs1203834
rs1230018
rs10822155
rs116528662
rs114694170
rs62640435
rs34536806
rs12784822
rs145846107
rs16883415
All − MR Egger
All − Inverse variance weighted
−2
−1
0
1
2
MR effect size for
'exposure' on 'outcome'
